# Contiguity of proactive and reactive inhibitory brain areas: a cognitive model based on ALE meta-analyses

**DOI:** 10.1007/s11682-020-00369-5

**Published:** 2020-08-03

**Authors:** Gioele Gavazzi, Fabio Giovannelli, Tommaso Currò, Mario Mascalchi, Maria Pia Viggiano

**Affiliations:** 1 IRCCS, SDN, Naples, Italy; 2 Department of Neuroscience, Psychology, Drug Research, Child Health, University of Florence, Florence, Italy; 3 Department of Experimental and Clinical Biomedical Sciences, University of Florence, Florence, Italy

**Keywords:** Proactive inhibition, Cognitive control, Meta-analysis, Right inferior frontal Gyrus, Right middle frontal Gyrus

## Abstract

Cognitive control is a critical feature in adapting our behavior to environmental and internal demands with two types of inhibition having been identified, namely the proactive and the reactive. Aiming to shed light on their respective neural correlates, we decided to focus on the cerebral activity before or after presentation of the target demanding a subject’s stop as a way to separate the proactive from the reactive components associated with the tasks. Accordingly, we performed three Activation Likelihood Estimation (ALE) meta-analyses of fMRI studies exploring proactive and reactive inhibitory phases of cognitive control. For this purpose, we searched for fMRI studies investigating brain activity preceding or following target stimuli. Eight studies (291 subjects, 101 foci) were identified for the proactive analysis. Five of these studies and those previously analyzed by others (348 subjects, 199 foci) were meta-analyzed to explore the neural correlates of reactive inhibition. Overall, our results showed different networks for the two inhibitory components. Notably, we observed a contiguity between areas in the right inferior frontal gyrus pertaining to proactive inhibition and in the right middle frontal gyrus regarding reactive inhibition. These neural correlates allow proposal of a new comprehensive model of cognitive control.

High order processes such as decision making involve adaptive and flexible cognitive control. This adaptability provides the resources to modify planned responses and to appropriately react to ever-changing information from external stimuli and internal goals (Duque et al. 2017).

Cognitive control operates through two inhibition mechanisms—proactive and reactive modes—depending on the time the action is withheld.

Proactive inhibition may lead to specific response tendencies and concerns the process of preparing actions to achieve a current target by facilitating, if necessary, the suppression of the impending action (Aron 2011). The proactive process is a ‘top-down’ form of control: by intervening before event occurrence, it enhances accuracy and efficiency of the motor response (Braver 2012). Reactive inhibition is a ‘bottom-up’ form and it is thought to be a “cut-trigger” stopping of an already initiated motor response (Meyer and Bucci 2016). Although both processes play a fundamental role in cognitive control to prevent maladaptive behaviours (Gazzaniga et al. 2014), the nature of their respective relationship remains a long-standing unanswered question.

Some studies on cognitive control have addressed the relationship between proactive and reactive mechanisms assuming a differential recruitment reflecting external demands. Other studies have viewed the two processes as opposite poles on a continuum (Chiew and Braver 2017). Admittedly, a general limitation of these studies is the difficulty to design paradigms capable of directly untangling the two processes.

As a consequence, knowledge regarding the neuroanatomical correlates of the two inhibitory processes is unbalanced. Indeed, although the ability to anticipate future events is the basis of any behavior, human behavior has been explained by investigating mainly reactive functions and their underpinning neural networks (see Bari and Robbins 2013 and Aron et al. 2014 for review).

Experimental evidence supports the view that the reactive inhibition process recruits a right-lateralized fronto-parietal circuit (e.g. Corbetta et al. 2008). In one such example, Simmonds et al. (2008) conducted a meta-analysis of neuroimaging studies to evaluate the brain areas involved in reactive control by gathering results from ten investigations that implemented a Go/No-go task during fMRI acquisitions. The studies were divided into two groups: “simple” and “complex”, depending on the working memory task demand. Overall, the meta-analysis revealed that right-lateralized prefrontal-parietal circuits were observed only in complex tasks, whereas the pre supplementary motor area (pre-SMA) was involved in response inhibition regardless of tasks complexity.

Conversely, limited literature is currently available on brain substrates of the proactive phase of inhibition, and no meta-analyses have considered neuroimaging studies. Proactive inhibition has recently been configured as a default modality of executive control, mediated by a sort of “braking-accelerator system” (Aron 2011; Criaud et al. 2012). More precisely, the top-down control (accelerator) would consist in a temporary release (braking) of the inhibitory default state of our motor system. Proactive inhibition seems to rely on a wide network that includes the pre-SMA, the subthalamic nucleus (STN), the right inferior frontal gyrus (rIFG) and the striatum (Cai et al. 2016; Aron et al. 2014), the prefrontal cortex, the inferior parietal cortex (Jaffard et al. 2008) and the primary motor cortex (Claffey et al. 2010; Sinclair and Hammond 2009).

As a matter fact, converging evidence shows that regions recruited both in proactive and reactive inhibition processes partially overlap (Chikazoe et al. 2009; Zandbelt and Vink 2010; Zandbelt et al. 2011; van Belle et al. 2014). These shared brain activations may hypothetically depend on intrinsic constraints of tasks that appear not well-suited for differentiating the two processes (Meyer and Bucci 2016). On the other hand, the overlapping activations might reflect functional or network sharing between the two inhibitory processes (Best et al. 2016). In an attempt to rule out intrinsic limits of behavioural tasks, we explored the two distinct phases of the inhibitory processes, assuming that volumes following a stimulus-triggering response correspond to the reactive phase, whereas the volumes preceding the stimulus are related to the proactive phase.

For this purpose, we conducted a meta-analysis, gathering data from studies which implemented fMRI data during behavioural tasks commonly employed to assess inhibitory processes (e.g. Stop Signal Task and the Go/No-go task). Admittedly, the double nature of these tasks does not help separate involvement of the two components from each other. However, by focusing exclusively on the timing of the cerebral activity (acquired volumes), i.e. before or following presentation of the target demanding the subject’s stop, separation of the proactive from the reactive components associated with the tasks would be possible. For the sake of completeness, we also included data originally collected by Simmonds et al. (2008) to account for differences due to technical and statistical updating of the ALE algorithm.

## Materials and methods

### Literature search and selection

We conducted a systematic and comprehensive literature search to select fMRI studies published up to January 2019 using the databases *PubMed* (https://www.ncbi.nlm.nih.gov/pubmed/) and *Web of Science* (https://webofknowledge.com). The selected keywords were combined using the Boolean operator AND and OR. The *PubMed* search input was: ((((proactive control) OR proactive inhibition) OR alerting) OR readiness) AND fMRI. The *Web of Science* search input was: TS = ((proactive control OR proactive inhibition OR alerting OR readiness) AND fMRI). Additional studies were searched from the references of all identified publications. Eligibility was determined by a two-step procedure performed by three of the authors (GG, FG, and TC). First, the titles and abstracts of all identified articles were screened. In the second step, the full texts of studies, according to predefined eligibility criteria, were independently examined and agreement was reached after discussion. Our study was conducted following the preferred reporting items for systematic reviews and meta-analyses (PRISMA) guidelines (Moher et al. 2009) (PRISMA checklist is provided in the Appendices Fig. 5 and Table 2).

The studies were included for the quantitative analyses if they met the following criteria: 1) whole-brain analysis performed on fMRI data (we excluded studies conducted by positron emission tomography and fMRI studies in which only results from ROI analysis were reported); 2) availability of coordinates of activation foci clearly provided either in Montreal Neurological Institute (MNI) or Talairach reference space; 3) availability of data related to the brain activity preceding a target stimulus (e.g. Go or No-go - defined as proactive, preparatory, warning, or readiness period); 4) availability of studies conducted on healthy participants reporting contrasts against rest or baseline. We excluded studies that used the go trials or other contrast conditions as baseline in the proactive phase. The selection of these strict criteria allowed us to select homogeneous studies in order to obtain theoretically more robust measures (Borenstein et al. 2009).

### Activation likelihood estimation (ALE)

Activation likelihood estimation (ALE) meta-analysis (Turkeltaub et al. 2002, 2012; Laird et al. 2005; Eickhoff et al. 2012) was performed using GingerALE 2.3.6 (www.brainmap.org/ale). Neuroanatomical coordinates reported in MNI space were transformed to Talairach space (Talairach et al. 1997).

ALE is a coordinate-based meta-analysis technique that uses peak coordinates reported in functional studies as input. The description of the exact procedure of ALE meta-analyses can be found in several methodological papers (Eickhoff et al. 2009, 2012) and herein we shall only summarize it. Controlling for the sample size, the ALE algorithm evaluates the convergence of activation foci from different neuroimaging studies, modeled as probability distributions of activation (Eickhoff et al. 2009) at given coordinates, against null distributions of random spatial associations between studies. Data were elaborated with the non-additive algorithm, described in Turkeltaub et al. (2012), to minimize within-experiment effects. Inference was made at cluster-level, as this procedure yields the better balance between sensitivity and specificity (Eickhoff et al. 2012) as compared to other methods. The cluster forming threshold was set at *P* < 0.005 (uncorrected at the voxel-level) and the size of the resulting supra-threshold clusters was compared (applying a threshold of *P* < 0.05) to a null distribution of cluster sizes determined by 1000 permutations of the data.

### Appendix 1 details the studies we selected

The first meta-analysis was conducted in order to include the activation foci generated in the contrasts associated with the volume preceding the stimulus against the baseline (Proactive Process). The second one included the activation foci generated in the contrasts associated to the volume following the stimulus against the baseline (Reactive Process: Nogo-Baseline) of our studies cumulated with those reported by Simmonds et al. (2008). The overlap among these two types of inhibition was analyzed by performing a conjunction analysis across the two processes. The differences in activation in the two inhibitory domains were identified by pairwise subtraction analyses (Eickhoff et al. 2012). We employed a statistical threshold of uncorrected *p* < 0.01 with 10,000 permutations and a cluster-size threshold of 200 mm3.

In the last analysis we rerun the data collected by Simmonds et al. (2008) dealing with the reactive process, that is, the foci generated in the contrasts associated to the volume following the stimulus against the baseline (“Nogo-Baseline”), using the algorithm adopted for the two above meta-analyses. In particular, in all meta-analyses we defined as baseline the activation measured from the volumes acquired in the rest time interval at the end of each trial.

Whole-brain maps of the thresholded ALE images were visualized in Mango V.4.0.1 (http://rii.uthscsa.edu/mango/) which is an anatomical image overlay program, and superimposed onto a standardized anatomical template in Talairach space.

## Results

Our search yielded 73 potentially eligible studies (the flow chart regarding article selection is illustrated in Appendices Fig. 5 and Table 2). After full-text assessment of these articles, we excluded studies not designed to evaluate the proactive component, studies reporting only ROI analysis results, and studies employing behavioral tasks aimed to assess conflict. Hence eight studies published from 2008 to 2018 were included in the quantitative analysis. From these eight studies, a cumulative number of 291 healthy subjects and 101 foci resulted.

Only five of the eight studies matched the same criteria adopted by Simmonds et al. (2008) to explore the reactive inhibitory component. Therefore, we included in the quantitative analysis those five studies resulting from our selection and the eleven studies previously gathered by Simmonds et al. (2008) for a total number of 348 healthy subjects and 199 foci. The main characteristics of the studies meta-analyzed are reported in Appendix Table 3.

In a separate quantitative analysis we also included the original eleven studies performed between 2000 and 2004 that were previously meta-analyzed by Simmonds et al. (2008) with a cumulative number of 212 healthy subjects and 140 foci on the reactive inhibitory phase alone.

**Fig. 1:**
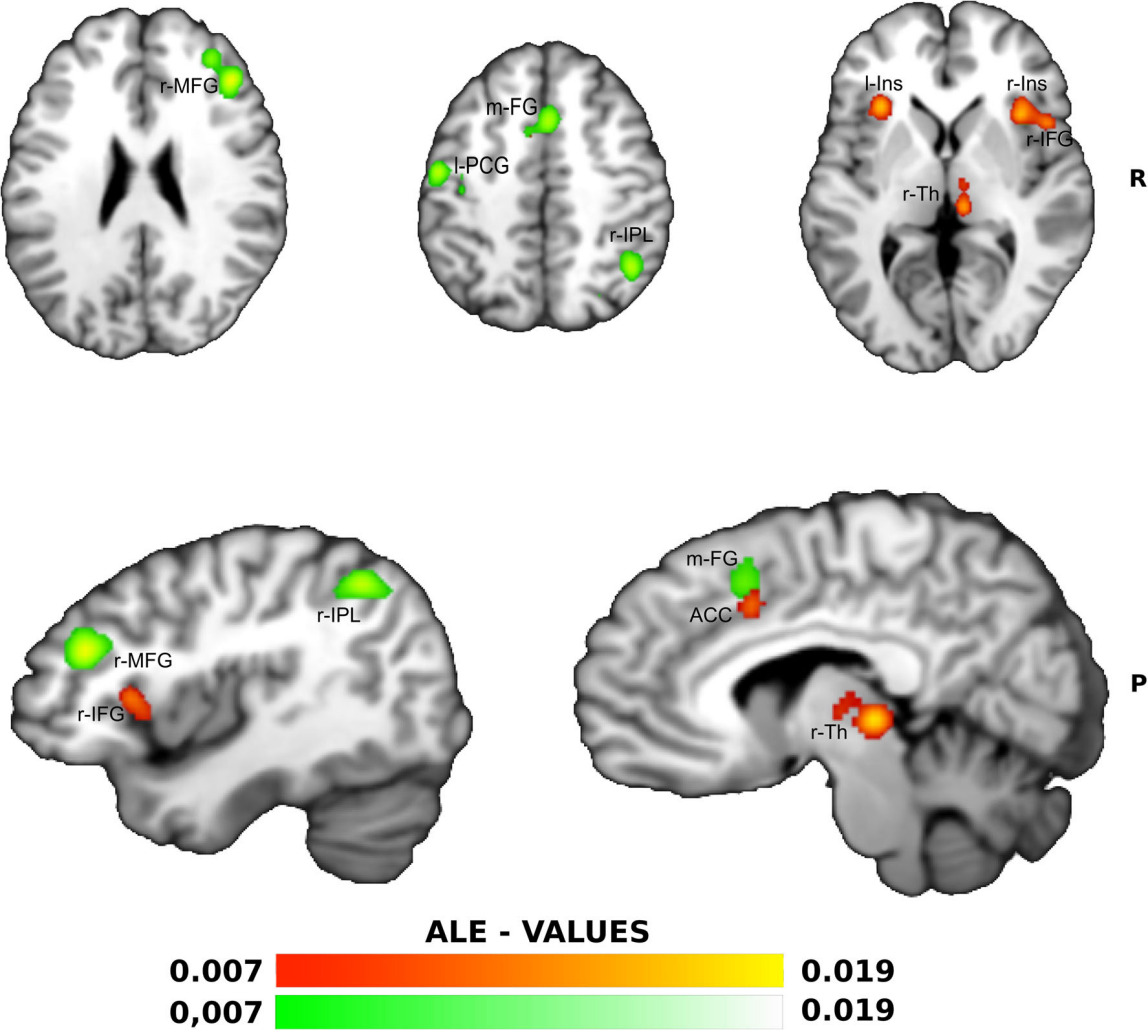
**ALE meta-analysis map for the Proactive and Reactive Inhibition process of our data selection.** The algorithm converged for Proactive process (in yellow-red) on right Insula (Ins) and extended to rIFG, left Ins, right Thalamus (Th) and bilaterally the Anterior Cingulate Cortex (ACC). The algorithm converged for Reactive process (in white-green) on the right Middle Frontal Gyrus (r-MFG), left Pre-Central Gyrus (l-PCG), medial Frontal Gyrus (m-FG) and right Inferior Parietal Lobule (r-IPL)- *P* < 0.05 cluster-level corrected inference using *P* < 0.005 uncorrected at voxel-level as the cluster-forming threshold

The ALE meta-analysis of the proactive inhibitory phase (Fig. 1) identified the largest size cluster (3064 mm^3) in the right insula with extension to the r-IFG, followed by the cluster in the left insula (1888 mm^3), the cluster in the right thalamus (1440 mm^3), and the cluster encompassing bilaterally the anterior cingulate cortex (ACC - 1120 mm^3).

In the analysis of the reactive phase (11 studies from Simmonds et al. 2008 combined with ours five out of eight compatible studies, Fig. 1), the algorithm identified the largest cluster in terms of size (3184 mm^3) in the right middle frontal gyrus (r-MFG) and superior frontal gyrus (r-SFG); a cluster (2616 mm^3) centered in the right inferior parietal lobule and precuneus and a cluster (2312 mm^3) located in the left pre-central gyrus followed. Another cluster (1856 mm^3) was included in the right insula and a last cluster (1712 mm^3) in the medial frontal gyrus (Tab 1, Fig. 1).

**Table 1:** **Results from ALE meta-analysis.** Foci are reported in Talairach coordinates. BA = Brodmann’s area

Proactive Process: ALE metanalysis computed from our study selection						
Cluster#	Vol. (mm^3)	Ext. Val	x	y	z	Label
1	3064	0.01867947	34	18	8	Right Cerebrum.Sub-lobar.Insula.Gray Matter.BA 13
		0.016100656	32	20	0	Right Cerebrum.Sub-lobar.Insula.Gray Matter.*
		0.013882096	46	14	4	Right Cerebrum.Sub-lobar.Insula.Gray Matter.BA 13
2	1888	0.017532656	−30	22	6	Left Cerebrum.Sub-lobar.Insula.Gray Matter.BA 13
		0.01677453	−28	16	−2	Left Cerebrum.Sub-lobar.Claustrum.Gray Matter.*
3	1440	0.019304343	8	−24	2	Right Cerebrum.Sub-lobar.Thalamus.Gray Matter.*
		0.010329299	8	−16	8	Right Cerebrum.Sub-lobar.Thalamus.Gray Matter.Medial Dorsal Nucleus
4	1120	0.01255372	−8	10	40	Left Cerebrum.Limbic Lobe.Cingulate Gyrus.Gray Matter.BA 32
		0.01195102	6	14	38	Right Cerebrum.Frontal Lobe.Cingulate Gyrus.Gray Matter.BA 32
		0.009141383	−6	6	46	Left Cerebrum.Limbic Lobe.Cingulate Gyrus.Gray Matter.BA 24
Reactive Process: ALE metanalysis computed from our and Simmonds et al. (2008) studies selection						
Cluster#	Vol. (mm^3	Ext. Val	x	y	z	Label
1	3184	0.020494524	42	34	24	Right Cerebrum.Frontal Lobe.Middle Frontal Gyrus.Gray Matter.BA 46
		0.014735842	32	44	24	Right Cerebrum.Frontal Lobe.Middle Frontal Gyrus.Gray Matter.BA 10
		0.01393399	36	24	34	Right Cerebrum.Frontal Lobe.Middle Frontal Gyrus.Gray Matter.BA 9
		0.010021554	28	52	36	Right Cerebrum.Frontal Lobe.Superior Frontal Gyrus.Gray Matter.BA 9
		0.0095843645	26	46	32	Right Cerebrum.Frontal Lobe.Superior Frontal Gyrus.Gray Matter.BA 9
2	2616	0.018389503	40	−52	44	Right Cerebrum.Parietal Lobe.Inferior Parietal Lobule.Gray Matter.BA 40
		0.01829336	48	−44	40	Right Cerebrum.Parietal Lobe.Inferior Parietal Lobule.Gray Matter.BA 40
		0.011250421	26	−64	38	Right Cerebrum.Parietal Lobe.Precuneus.Gray Matter.BA 7
3	2312	0.017564192	−50	−10	46	Left Cerebrum.Frontal Lobe.Precentral Gyrus.Gray Matter.BA 4
		0.015164279	−38	−18	48	Left Cerebrum.Frontal Lobe.Precentral Gyrus.Gray Matter.BA 4
		0.0087818615	−38	−10	44	Left Cerebrum.Frontal Lobe.Precentral Gyrus.Gray Matter.BA 6
4	1856	0.015462176	36	18	4	Right Cerebrum.Sub-lobar.Insula.Gray Matter.BA 13
		0.0137887085	36	10	−2	Right Cerebrum.Sub-lobar.Insula.Gray Matter.BA 13
5	1712	0.018349858	2	16	44	Right Cerebrum.Frontal Lobe.Medial Frontal Gyrus.Gray Matter.BA 6
		0.010666837	−8	10	44	Left Cerebrum.Frontal Lobe.Medial Frontal Gyrus.Gray Matter.BA 32
Reactive Process: ALE metanalysis computed from Simmonds et al. (2008) selection						
Cluster#	Vol. (mm^3)	Ext. Val	x	y	z	Label
1	2504	0.020474896	42	34	24	Right Cerebrum.Frontal Lobe.Middle Frontal Gyrus.Gray Matter.BA46
		0.013930568	36	24	34	Right Cerebrum.Frontal Lobe.Middle Frontal Gyrus.Gray Matter.BA9
2	1736	0.014614802	−16	2	6	Left Cerebrum.Sub-lobar.Lentiform Nucleus.Gray Matter.Putamen
3	1408	0.017052285	−42	−62	−12	Left Cerebrum.Temporal Lobe.Fusiform Gyrus.Gray Matter.BA37
4	1384	0.014383861	−44	−40	40	Left Cerebrum.Parietal Lobe.Inferior Parietal Lobule.Gray Matter.BA 40

**Fig. 2:**
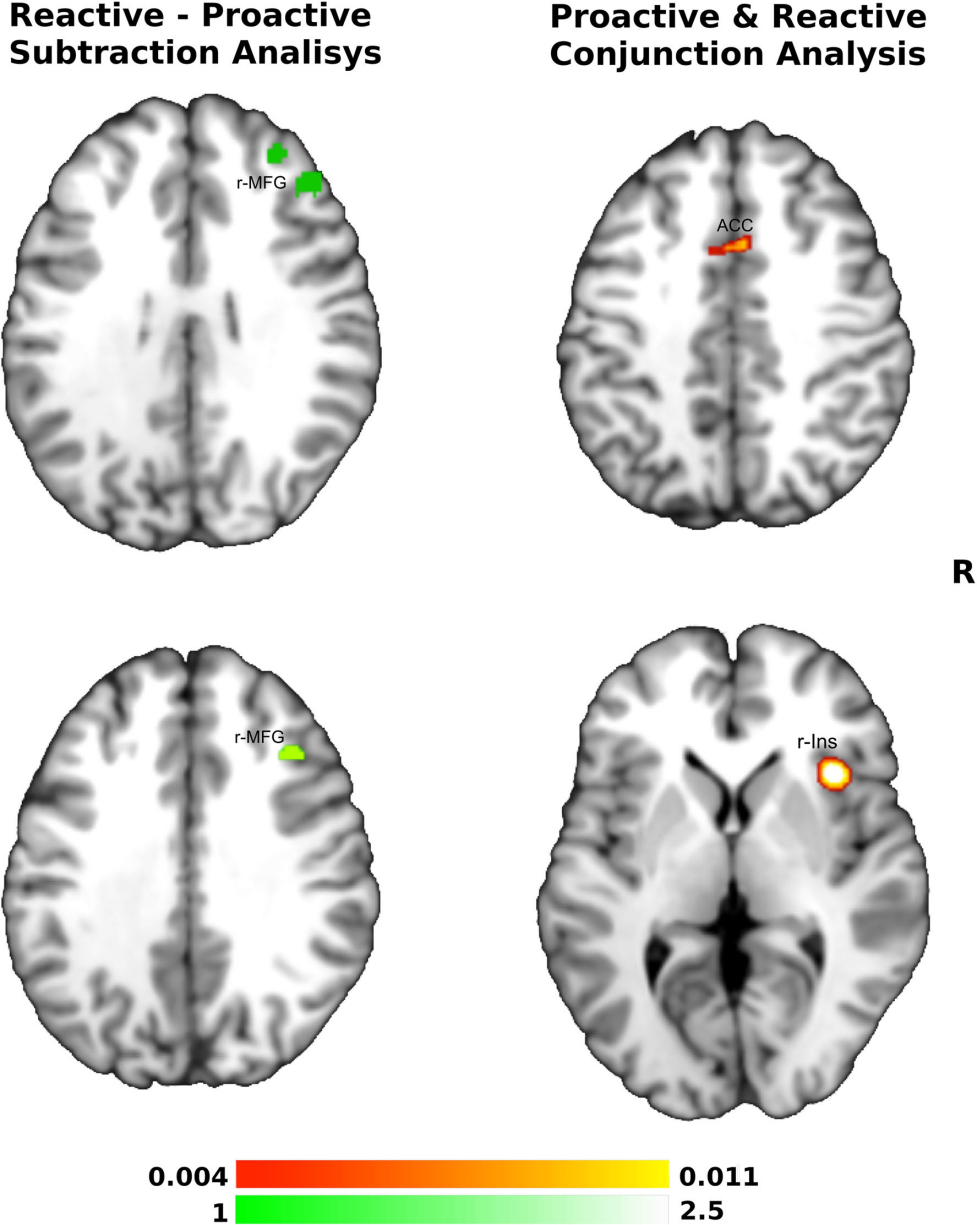
**Conjunction and subtraction analyses of Proactive and Reactive Inhibitory process.** The scale bar in red represents minimum ALE values from 0.004 to 0.011 in the conjunction analysis - Anterior Cingulate Cortex (ACC) and right Insula (r-Ins) activations. The scale bar in green represents z-values from 1 to 2.5 revealed by the contrast Reactive Inhibition > Proactive inhibition (right Middle Frontal Gyrus, r-MFG)

In the conjunction analysis of these two inhibitory phases, we found common activation (as shown in yellow-red in Fig. 2) in the right insula (960 mm^3) and ACC (288 mm^3). Direct contrast between proactive and reactive inhibitory phases revealed higher convergence of activity for the reactive inhibition (as shown in white-green in Fig. 2) in the r-MFG (1672 mm^3).

**Fig. 3:**
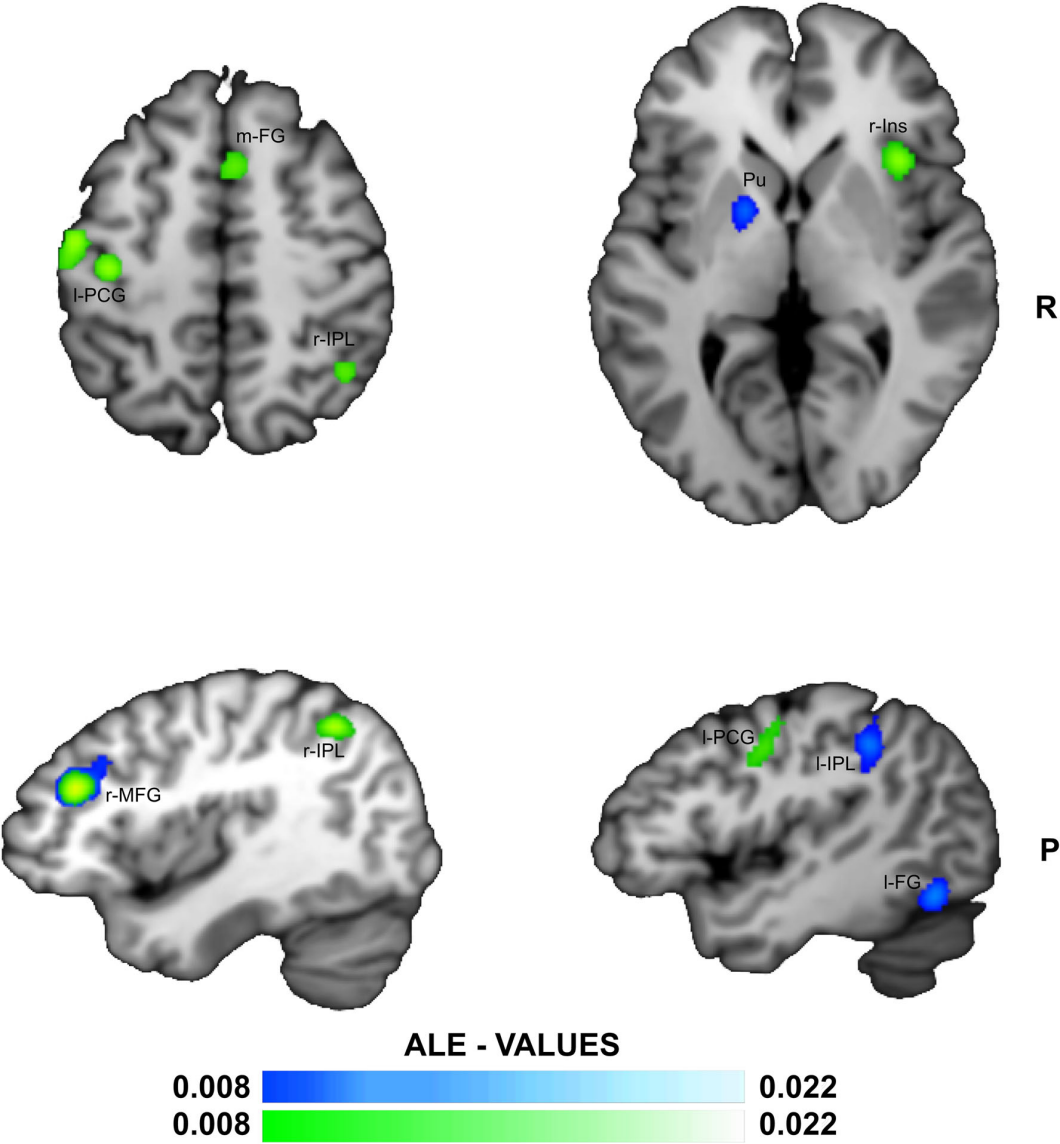
**ALE meta-analysis map for the Reactive Inhibition process.** The algorithm converged for our data cumulated with Simmonds et al. (2008) on the right Middle Frontal Gyrus (r-MFG), left Pre-Central Gyrus (l-PCG), medial Frontal Gyrus (m-FG) and right Inferior Parietal Lobule (r-IPL), as shown in white-green. The algorithm converged for reanalysis of Simmonds et al. (2008) data alone (in white-blue) on the right Middle Frontal Gyrus (r-MFG), the left Inferior Parietal Lobule (l-IPL), left Frontal Gyrus (l-FG) and the left Putamen (Pu) - *P* < 0.05 cluster-level corrected inference using *P* < 0.005 uncorrected at voxel-level as the cluster-forming threshold

Re-analysis of the data collected by Simmonds et al. (2008) with the updated algorithm yielded significant convergence of activation in four clusters (Fig. 3) that were centered in the right middle frontal gyrus (2504 mm^3), in the left putamen (1736 mm^3), in the left fusiform gyrus (1408 mm^3), and in the left inferior parietal lobule (1384 mm^3).

## Discussion

Since the earliest studies on cognitive control, researchers have focused on the investigation of proactive and reactive inhibitory processes. So far, all meta-analyses exploring with neuroimaging the neural correlates of inhibitory processes have been conducted on the reactive inhibitory phase. The present work reports for the first time a meta-analysis carried out by gathering data from studies that explored both the proactive and reactive components, mainly the Stop Signal Task (SST) and the Go/No-go task acquired during fMRI. The ALE meta-analysis of foci associated with the proactive inhibition phase showed significant convergence of activation in bilateral ACC and insulae, the rIFG and right thalamus.

As previously reported in studies on proactive inhibition (Kinomura et al. 1996; Yanaka et al. 2010; Gavazzi et al. 2018a, b) insulae could absolve functions associated with the alerting and salience processes (Taylor et al. 2009; Eckert et al. 2009). Along this line, also involvement of the right thalamus is consistent with the view that an alerting process is necessary to keep proactive inhibition engaged. In fact, the intra-thalamic nuclei are engaged to maintain a state of high vigilance or alerting (Kinomura et al. 1996; Yanaka et al. 2010). On the other hand, there is neuroimaging and neuropsychological convergence evidence that suggests rIFG plays a pivotal role in inhibitory processes across a range of tasks, requiring suppression of response tendencies (Aron et al. 2003, 2014).

The ACC may ultimately be the key region in the proactive network. Indeed, the ACC is ideally suited as a crucial brain region for switching the proactive inhibitory modality into the reactive inhibitory control. In fact, many studies have shown that ACC monitors the information from incoming external stimuli in search of conflicts (Bari and Robbins 2013; Botvinick et al. 2001), rather than resolving them (Kerns 2004; Botvinick et al. 2004). Moreover, a nonparametric voxel-based lesion-symptom mapping performed in a large sample of patients with focal brain lesions (Glascher et al. 2012) supports the view that the key function of the ACC (particularly the rostral part) may be set shifting, whereas other regions including the right pre-frontal cortex may be involved in error detection as well as inhibitory functions.

Proactive inhibition has been considered a default modality of executive control, mediated by a sort of ‘braking-accelerator system’ (Criaud et al. 2012). In a similar way, the insulae with the right thalamus and the r-IFG could represent the core of the proactive inhibitory network operating in a continuous balance between the inhibitory component, exerted by the r-IGF, and the excitatory component engaging the insulae and the thalamus (part of the alertness network). The shift from proactive to reactive inhibitory status would thus be determined by recruitment of the ACC. In this network, the ACC would allow income of external stimuli, evaluation of their saliency, and maintenance of information online. Notably, several studies support communication between the cerebral regions characterizing the braking-accelerator system (insulae, right thalamus and r-IFG) and the ACC. Cai et al. (2014) documented a functional connectivity between the ACC and right anterior insula, representing a node of a system that facilitates detection and attention to salient events (Seeley et al. 2007; Menon and Uddin 2010). In particular, the right anterior Insula would have the role of detecting potentially or behaviorally significant events, whereas the ACC would update internal attention settings in response to those events (Han et al. 2019).

The ALE meta-analysis we performed on data related to the reactive inhibition phase in the same studies exploited for proactive inhibition along with those gathered by Simmonds et al. (2008) showed significant convergence of activation in the r-MFG, medial/superior frontal gyrus, inferior parietal lobule, precuneus, insula and in the left precentral gyrus. Interestingly, when we repeated the ALE meta-analysis with the updated ALE algorithm of the foci associated with the reactive inhibition phase of the data collected by Simmonds et al. (2008), significant convergence of activation in right middle frontal gyrus, right putamen, left fusiform gyrus and left inferior parietal lobule emerged. Thus, only r-MFG was consistent between the two meta-analyses (Fig. 3). This is noteworthy since we followed the same criteria as Simmonds et al. (2008) to select the data to be analyzed, with the updated algorithm being the only difference between the two analyses. In their original analysis of reactive inhibition foci (see Appendix Table 4), Simmonds et al. (2008) observed the pre-SMA, right pre-frontal regions, (r-IFG and r-MFG) left pre-motor cortex, bilateral inferior parietal regions, insulae, putamen and occipital regions.

Thus, our re-analysis allows us to address two main points.

First, both phases of inhibitory control (proactive and reactive) were included in the list of brain regions reported by Simmonds et al. (2008), although their meta-analysis was based exclusively on reactive inhibition (namely, volumes following the stimulus). In particular, by comparing our results (pre-stimulus and post-stimulus volumes) with those reported by Simmonds et al. (2008) it becomes possible to map the brain networks involved in the two components of the inhibitory process. Except for the ACC and right thalamus, all brain regions revealed by the studies we selected are almost comprised in those (see Appendix Table 4) reported by Simmonds et al. (2008). Such discrepancies would suggest that insulae and r-IFG, which we observed only in the proactive inhibitory process, may have been reported in the reactive process by Simmonds et al. (2008) due to methodological and statistical parameters differences. Indeed, several studies have shown how previous ALE algorithms were prone to statistical error type I (e.g. Eickhoff et al. 2012), leading to false positive results. However, this mismatch could result from the higher type I error ratio of the decade-old algorithm in ALE meta-analyses and/or the improved mapping of BOLD signal over the last ten years (we included studies after 2008). Future investigations are necessary to address this point.

Second, r-MFG was the only region related to the reactive process to consistently emerge among the meta-analyses conducted in the present study, in particular considering the contrast between the reactive and the proactive inhibition (Fig. 2). Differently, according to the interpretation by Simmonds et al. (2008), pre-SMA plays a primary reactive role regardless of task complexity. Indeed, pre-SMA has been reported as involved in interrupting an ongoing action (Swann et al. 2013; Picazio et al. 2014), whereas r-IFG seems to play an extensive role in inhibitory functioning (Aron et al. 2003, 2014). This latter has been corroborated by Gavazzi et al. (2018b) who described a patient with damage to almost the entire right hemisphere during puberty who subsequently recovered, or preserved, her inhibitory functions by engaging the left homotopic IFG region exclusively, but not the pre-SMA.

Overall, our analyses make it possible to spatially differentiate areas involved in proactive and reactive components of inhibition which formerly were assigned to a single network (see Appendix Table 4). Notably, we have shown that the inhibitory component involved in proactive processes mainly recruits the r-IFG, whereas the inhibitory component employed in reactive processes exclusively engages the r-MFG.

**Fig. 4:**
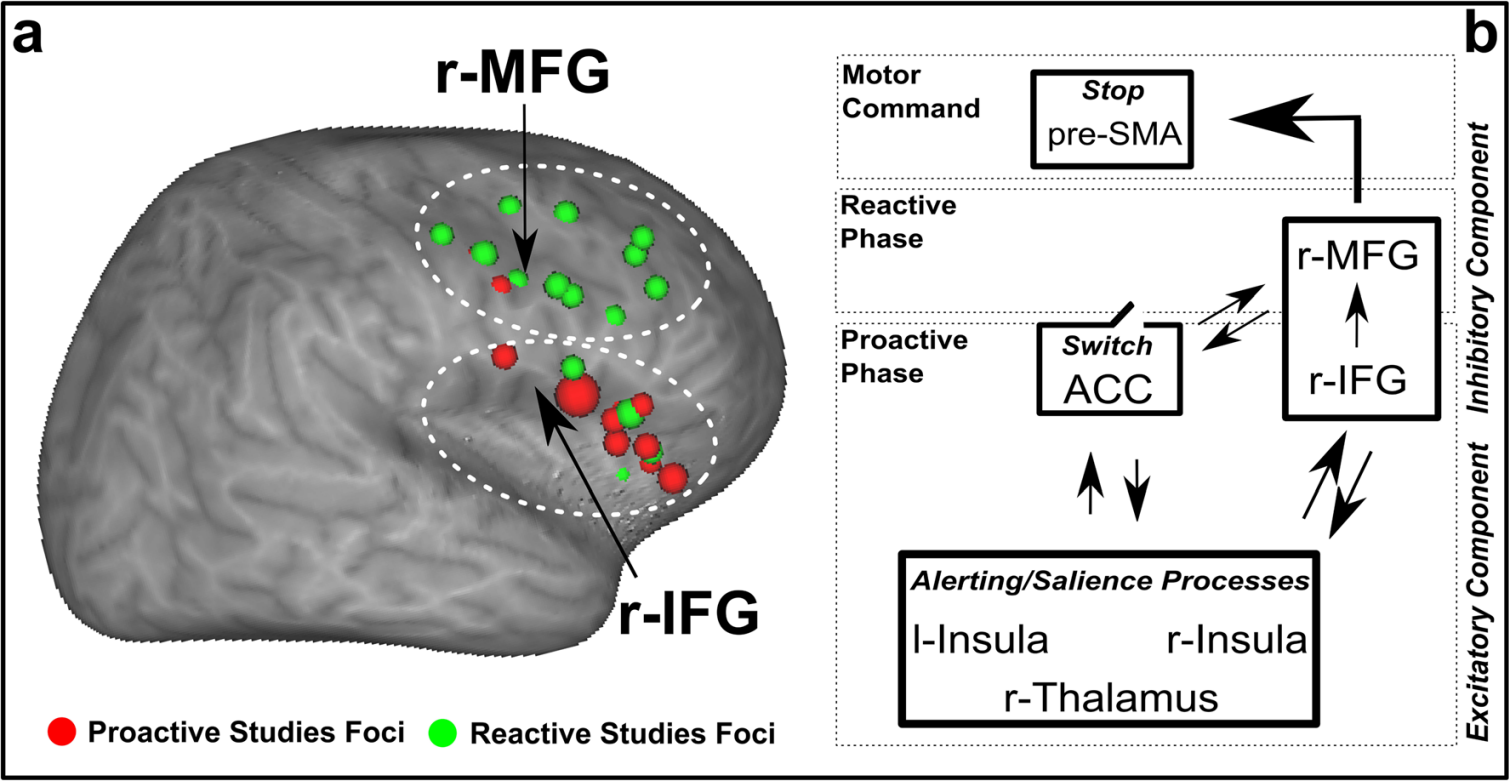
**Contiguity in right pre-frontal cortex between proactive (r-IFG) and reactive (r-MFG) inhibitory processes. a** - Foci meeting criteria for inclusion in the metanalysis of proactive (red sphere) and reactive (green sphere) inhibitory processes. Each foci is represented by a sphere with size proportional to the number of subjects enrolled in the study. From top to down, the two dotted white lines identify the r-MFG and the r-IFG brain regions, respectively. **b** - This panel illustrates the Excitatory, Inhibitory and Switch components of the P-R M. According to the model the excitatory component would be exerted by the thalamus and both Insulae, whereas the inhibitory component would recruit the r-IFG and the r-MFG for the proactive and reactive inhibitory processes, respectively. The ACC would play the role of the switch component turning the proactive network into the reactive one

The anatomical contiguity of the two cortical regions involved in different phases (r-IFG for proactive and r-MFG for reactive) of the inhibitory control is in line with the distributed nature of the brain systems associated with the higher functions in mammals (Fig. 4a). This distributed nature may be associated, on the one hand, with connections between cortical association areas in the transverse cortical dimension, and, on the other hand with the columnar organization of the neocortex (e.g. Mountcastle 1997) that underpins the reciprocal linkage between cortical and subcortical structures. For example, columnar organization has been demonstrated in the frontal association cortex, namely Brodmann areas 46 and 9, in the dorsolateral prefrontal cortex (see Petrides 2005).

**Fig. 5:**
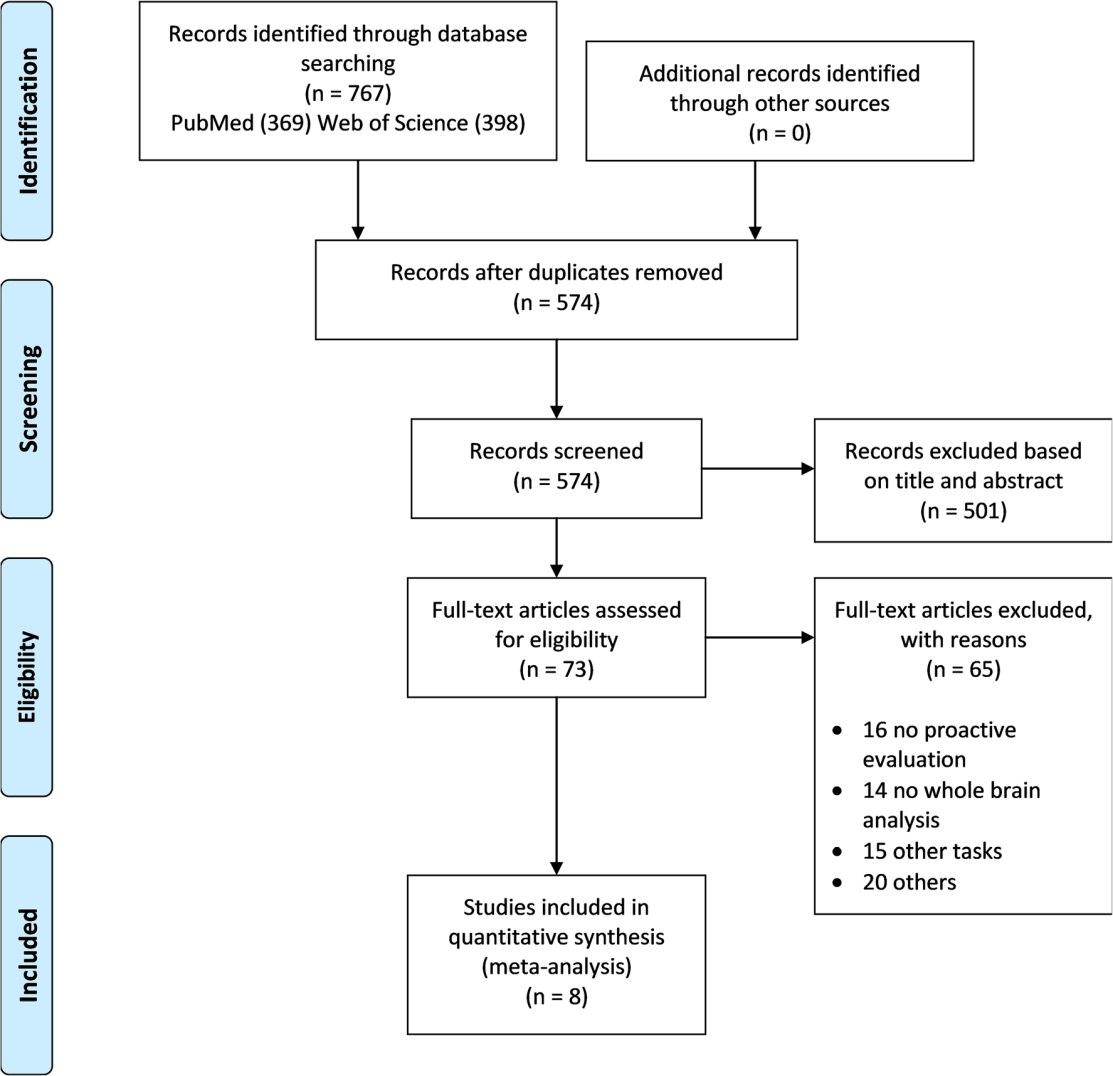
PRISMA flow chart of study selection

The right inferior cortices and pre-SMA are traditionally considered key structures in the inhibitory component of cognitive control (e.g. Aron et al. 2003). According to our results, it seems that we might re-define the roles of these structures.

As we observed the r-IFG only in the proactive network, but neither in the reactive network nor in the shared network between proactive and reactive phases, this region might be viewed as a precursor of the reactive inhibitory process. Accordingly, the fact that several studies in human patients with damaged r-IFG justified attribution of the r-IFG to inhibitory control, it is not per se inconsistent with a hierarchically earlier/higher role in the general inhibitory process of this area. Moreover, our results confirm that the right middle frontal gyrus belongs entirely to the reactive inhibitory network.

On the basis of neural correlates revealed by our meta-analyses, we propose a new cognitive model to describe motor inhibitory processes (see Fig. 4b) which we have called the Proactive-Reactive Model (P-R M).

Cognitive control of our actions relies on proactive and reactive phases that operate in synergy, optimizing environmental interactions. In our view, the proactive process is based on an Excitatory, an Inhibitory and a Switch component (See Fig. 4b). The Excitatory component requires thalamus and insulae activations (as discussed above), whereas r-IFG would play the role of an Inhibitory component in order to balance the forces. For its specific functions, the ACC may play the role of the Switch component, turning the reactive phase into a proactive one. In fact, ACC does not only form a communication node for saliency with the right insula and borders with r-MFG, but it also is the key region in monitoring and selecting conflictual stimuli coming from the external world (e.g. MacDonald 2000). Notably ACC and the right insula are the only brain regions belonging both to proactive and reactive networks (Fig. 2). Therefore, as the main brain region capable of absolving all these functions, ACC is a good candidate for the role of Switch component. The reactive phase, on the other hand, seems to be controlled by r-MFG which in turn, depending on the specific request of the external environment (or the task in an experimental setting), recruits the necessary additional areas. This would explain why the r-MFG is exclusively and consistently found in all analyses focused on the reactive phase. Notably, in the re-run of the Simmonds et al. (2008) metanalysis, we did not find pre-SMA as a part of the inhibitory reactive network. However in light of the considerable literature supporting its involvement in reactive inhibition, we submit that this brain region absolves the specific function to stop the action. Indeed, pre-SMA is often reported in studies where the system is highly stressed, for instance those employing the Stop Signal Task, but rarely in Go/No-go protocols (see studies reported in Appendix Table 3).

Importantly, as mentioned above, the contiguity between r-IFG and r-MFG and their shared columnar organization strongly support the idea that the core of inhibitory component of the model resides in these two regions with common cytoarchitecture, whereas pre-SMA, having a different cytoarchitecture and being also nearer to the motor areas, seems to be more engaged in sending the motor command of stop. In other words, according to our model the inferior frontal cortices would communicate with the ACC both in proactive (r-IFG) and reactive (r-MFG) phases in order to evaluate when, and if, to inhibit an action. Consistently with other works (Swann et al. 2013; Picazio et al. 2014), the role of pre-SMA would be limited to providing the actual stop of motor commands.

By meta-analyzing neural correlates reported in task-based fMRI studies we propose a new model of cognitive control. Since all studies selected were based on fMRI protocols, we are aware that the accuracy of our results might be influenced by the low temporal resolution of this instrument. This is the main limitation of the present work. In particular, we recognize that the hypothesized role of switch we attributed to the ACC being temporally brief and spread between the proactive and reactive phases needs to be evaluated by further investigations. Different techniques with improved temporal resolution as compared to fMRI, like MEG and/or the combination of EEG-fMRI, might be used for this purpose with an exploration at higher temporal resolution by means of additional parameters of the BOLD contrast and beyond. With these methods one may verify the model proposed here as well as validate and potentially detail the mechanism of the ACC.

Moreover, on clinical grounds, a separate analysis of the proactive and reactive components in future fMRI studies might increase our understanding of addictive behaviors in psychiatric conditions and of the physiopathological mechanisms underlying loss of cognitive control in neurodegenerative or infective diseases predominantly affecting the frontal and cingulate cortices. These include, on the one hand, fronto-temporal dementia, Huntington chorea, and Progressive Supranuclear Palsy and, on the other hand, Herpes Encephalitis. The same approach might be pursued in vascular or inflammatory diseases involving the subcortical white matter as in small vessel diseases or multiple sclerosis. Finally, useful information could be gathered in cases of infarct in the territory of the anterior cerebral arteries which typically includes the ACC.

## Conclusion

We designed and performed meta-analyses on proactive and reactive inhibition by mirroring and replicating the study on reactive inhibition conducted a decade ago by Simmonds et al. (2008). We analyzed exclusively studies contrasting pre-stimulus volumes to explore proactive inhibition and post-stimulus volumes to investigate reactive inhibition brain regions. Our results have shown almost completely different networks for proactive and reactive processes and have allowed us to re-define the role of brain regions in the right frontal cortex. Based on these findings, we propose the P-R Model of cognitive control of actions, which has a proactive phase based on an excitatory component exerted by insulae and red thalamus balanced by an inhibitory component driven by the r-IFG. The shift of a proactive into a reactive phase would involve the ACC which operates as a sort of switch by analyzing external information and communicating with the right prefrontal cortices (namely r-IFG and r-MFG). Finally, the reactive phase would critically involve r-MFG which, depending on the specific task, would activate pre-SMA in order to stop actions.

## Appendix

**Table 2:** PRISMA 2009 Checklist

Section/topic	#	Checklist item	Reported on page #
Title			
Title	1	Identify the report as a systematic review, meta-analysis, or both.	1
Abstract			
Structured summary	2	Provide a structured summary including, as applicable: background; objectives; data sources; study eligibility criteria, participants, and interventions; study appraisal and synthesis methods; results; limitations; conclusions and implications of key findings; systematic review registration number.	2
Introduction			
Rationale	3	Describe the rationale for the review in the context of what is already known.	3–4
Objectives	4	Provide an explicit statement of questions being addressed with reference to participants, interventions, comparisons, outcomes, and study design (PICOS).	4–5
Methods			
Protocol and registration	5	Indicate if a review protocol exists, if and where it can be accessed (e.g., Web address), and, if available, provide registration information including registration number.	*Not available*
Eligibility criteria	6	Specify study characteristics (e.g., PICOS, length of follow-up) and report characteristics (e.g., years considered, language, publication status) used as criteria for eligibility, giving rationale.	5–6 and Appendix 2
Information sources	7	Describe all information sources (e.g., databases with dates of coverage, contact with study authors to identify additional studies) in the search and date last searched.	5
Search	8	Present full electronic search strategy for at least one database, including any limits used, such that it could be repeated.	5
Study selection	9	State the process for selecting studies (i.e., screening, eligibility, included in systematic review, and, if applicable, included in the meta-analysis).	5–6 and Appendix 1
Data collection process	10	Describe method of data extraction from reports (e.g., piloted forms, independently, in duplicate) and any processes for obtaining and confirming data from investigators.	5
Data items	11	List and define all variables for which data were sought (e.g., PICOS, funding sources) and any assumptions and simplifications made.	6–7
Risk of bias in individual studies	12	Describe methods used for assessing risk of bias of individual studies (including specification of whether this was done at the study or outcome level), and how this information is to be used in any data synthesis.	*Not available*
Summary measures	13	State the principal summary measures (e.g., risk ratio, difference in means).	6–7
Synthesis of results	14	Describe the methods of handling data and combining results of studies, if done, including measures of consistency (e.g., I^2^) for each meta-analysis.	6–7
Risk of bias across studies	15	Specify any assessment of risk of bias that may affect the cumulative evidence (e.g., publication bias, selective reporting within studies).	*Not available*
Additional analyses	16	Describe methods of additional analyses (e.g., sensitivity or subgroup analyses, meta-regression), if done, indicating which were pre-specified.	*Not reported*
Section/topic	#	Checklist item	Reported on page #
Results			
Study selection	17	Give numbers of studies screened, assessed for eligibility, and included in the review, with reasons for exclusions at each stage, ideally with a flow diagram.	7 and Appendix 1
Study characteristics	18	For each study, present characteristics for which data were extracted (e.g., study size, PICOS, follow-up period) and provide the citations.	Appendix 2
Risk of bias within studies	19	Present data on risk of bias of each study and, if available, any outcome level assessment (see item 12).	*Not available*
Results of individual studies	20	For all outcomes considered (benefits or harms), present, for each study: (a) simple summary data for each intervention group (b) effect estimates and confidence intervals, ideally with a forest plot.	Appendix 2
Synthesis of results	21	Present results of each meta-analysis done, including confidence intervals and measures of consistency.	8–9 and Table 1, Figs. 1-4
Risk of bias across studies	22	Present results of any assessment of risk of bias across studies (see Item 15).	*Not available*
Additional analysis	23	Give results of additional analyses, if done (e.g., sensitivity or subgroup analyses, meta-regression [see Item 16]).	*Not reported*
Discussion			
Summary of evidence	24	Summarize the main findings including the strength of evidence for each main outcome; consider their relevance to key groups (e.g., healthcare providers, users, and policy makers).	9–10
Limitations	25	Discuss limitations at study and outcome level (e.g., risk of bias), and at review-level (e.g., incomplete retrieval of identified research, reporting bias).	14
Conclusions	26	Provide a general interpretation of the results in the context of other evidence, and implications for future research.	15
Funding			
Funding	27	Describe sources of funding for the systematic review and other support (e.g., supply of data); role of funders for the systematic review.	16

From: Moher et al. (2009)

For more information, visit: www.prisma-statement.org

**Table 3:** Characteristics of studies included in the analysis

Study	Year	Sample size			fMRI Task	Proactive		Reactive	
		Number	Age (mean, SD or range)	Gender (f/m)		Contrast	Foci	Contrast	Foci
Jaffard et al.	2008	14	23–37	0/14	Event-related simple warned reaction time: subjects were instructed to respond as fast as possible by a button press once they detected the target regardless of whether it was preceded by a warning signal or not. For warned trials, the cue was presented with a variable delay (between 1100 and 1600 ms). Duration was about 38 mins. Data were analyzed with GLM	Cue vs Baseline	3	N.D	N.D.
Yanaka et al.	2010	27	24.1 ± 2.3; 22.8 ± 3.4	13/14	Event-related Go/NoGo task. The subjects were initially presented with a central fixation cross. After a relatively long ITI of 12–14 s, the color of the fixation cross changed from white to yellow as a warning stimulus. Following a variable time period (2–6 s), a blue or red square was presented as the Go signal or NoGo signal, respectively. When the Go signal was presented, the subjects had to respond by pressing a button with their right thumb as quickly as possible. Duration was about 30 mins. Data were analyzed with GLM	Warning, vs “rest”	10	NoGovsGo	5
Zandbelt et al.	2013	22	23,5 (20–28)	13/9	Event-related delayed-response version of the stop-signal anticipation task: participants are instructed to respond when a moving indicator reaches a target, but to suppress a response when this moving indicator stops automatically before reaching this target. Stop-signal probability was was 0%, 24%, or 35%.Duration was about 42 mins. Data were analyzed with GLM.	Cue (0%, 24%, 35%) vs Baseline (rest)	21	NoGo/baseline (rest)	7
Hu et al.	2015	114	30.7 ± 11.0	64/50	Event-related SST: go and stop trials were randomly intermixed in presentation with an inter-trial-interval of 2 s. A fixation dot appeared on screen to signal the beginning of each trial. After a fore-period varying from 1 s to 5 s, the dot became a circle – the “go” signal – prompting participants to quickly press a button. The circle disappeared at button press or after 1 s if the participant failed to respond. Duration was about 40 mins. Data were analyzed with GLM.	Unsigned prediction error	8	N.D.	N.D.
Bloemendaal et al	2016	48	25 young (mean age: 22.7 years, 18–29); 23 older adults (mean age: 67.6 years, range 61–74)	20/28	Event-related Load-Dependent Stop-Signal Anticipation Task. Information load increased with level. Stop-signal probability increased as a function of cue color. Every level contained 70 trials with 0% (green) and 270 trials with >0% (white) stopsignal probability. Duration was about 38 mins. Data were analyzed with GLM.	Cue volumes	19	StopSuccess > Go	13
Brevers et al.	2017	14	26,87*	10/6*	Event-related Stop signal task variant: participants had to discriminate between neutral and poker-related pictures. Subjects were asked to stop their response when they heard a tone (stop-signal). Four different cue provided stop signal probability, 0% (green), 17% (yellow), 25% (orange), and 33% (red); Duration was about 40 mins. Data were analyzed with GLM	Warning stimuli	26	N.D.	N.D.
Gavazzi et al.	2019	16	38.3 ± 11.0	8/8	Event-related Go/Nogo task: participants were asked to press a button as quickly as possible with their right index finger when a “Go” stimulus was presented and not to respond when “Nogo” stimulus was displayed. A descending series of asterisks was presented at the beginning of each trial to prepare participants to the proper GNG stimulus (“readiness” period).Duration was about 12 mins. Data were analyzed with GLM.	Warning stimuli vs Baseline (rest)	5	NoGo/baseline (rest)	14
Gavazzi et al	2018a	36	30.7 ± 6.6	21/15	Event-related Go/Nogo task: participants were asked to press a button as quickly as possible with their right index finger when a “Go” stimulus was presented and not to respond when “Nogo” stimulus was displayed. A descending series of points was presented at the beginning of each trial to prepare participants to the proper GNG stimulus (“readiness” period). Duration was about 12 mins. Data were analyzed with GLM.	Warning stimuli vs Baseline (rest)	9	NoGo/baseline (rest)	20

*Data provided for the entire control group, 2 subjects were excluded from analysis

**Table 4:** Results from ALE meta-analysis. Foci are reported in Talairach coordinates. BA = Brodmann’s area

Reactive Process: Original ALE metanalysis of all studies reported in Simmonds et al. (2008)							
Region	Hem	BA	x	y	z	Vol (mm3)	ALE (×10–3)
Inferior/Middle frontal gyrus	R	9/44	40	30	26	7464	9,21
Inferior parietal lobule	R	40	38	−50	42	6808	8,14
Superior medial wall (pre-SMA)	B	6/32	2	18	40	3712	7,95
Putamen/insula	L		−16	0	8	2624	6,03
Inferior parietal lobule	L	40	−44	−42	42	1784	5,88
Fusiform gyrus/posterior cerebellum	L	19/37	−40	−60	−14	1376	5,99
Middle occipital gyrus	R	19	44	−72	- 4	1032	4,67
Middle frontal gyrus	R	10	36	50	4	1016	5,19
Middle frontal gyrus	L	6	−40	8	42	368	4,35
Putamen/insula	R		32	16	0	280	4,12
Superior frontal gyrus	R	9	24	50	30	128	4,07
